# The long-term clinical impact of iliofemoral deep vein thrombosis

**DOI:** 10.1016/j.jvsv.2026.102551

**Published:** 2026-06-06

**Authors:** Nicos Labropoulos, Anisha Javvaji, Antonios Gasparis, Suresh Vedantham

**Affiliations:** a Division of Vascular and Endovascular Surgery, Stony Brook Medicine, Stony Brook, NY; b Renaissance School of Medicine, Stony Brook, NY; c Department of Surgery, Northwell Health, Greenlawn, NY; d Mallinckrodt Institute of Radiology, Washington University, St. Louis, MO

**Keywords:** Iliofemoral thrombosis, Long-term clinical outcomes, Post-thrombotic syndrome, Reflux, Obstruction

## Abstract

**Objective:**

Current literature shows a general lack of specificity and uniformity when objectively evaluating the long-term effects of iliofemoral deep vein thrombosis (IFDVT). The main purpose of our study was to follow the natural history of an IFDVT, treated with anticoagulation alone, in patients with a minimum of 5-year follow-up.

**Methods:**

This study included patients with a minimum of 5-year follow-up treated with anticoagulation alone. Patients who underwent additional interventional treatments, those with previous episode of deep vein thrombosis (DVT), immobility, severe comorbidities, systemic organ failures, chronic venous disease, and lymphedema were excluded. Bilateral lower limb ultrasound study was performed, and the limbs without DVT were used as a control group to compare outcomes. Every patient had clinical examination and a detailed duplex ultrasound guidance to evaluate reflux, obstruction, and recanalization at follow-up.

**Results:**

A total of 34 patients (68 limbs) were included in the study. Of these, 38 limbs had IFDVT and comprised the study group. Patients had a mean age of 49 ± 11 years. Of the limbs studied, 84.2% exhibited edema, 26% had skin damage, and three limbs presented with venous ulcers during follow-up. Venous claudication was observed in 18.4% of limbs, exclusively associated with multisegmented DVT. Ultrasound findings revealed 34.2% of the limbs had persistent obstruction. Reflux and obstruction were present in 57.9% of limbs with IFDVT. Limbs with both reflux and obstructions were significantly more likely to present with symptoms compared to limbs with either reflux or obstruction alone. The affected limbs were significantly more likely to have signs of post-thrombotic syndrome when compared with the contralateral unaffected limbs.

**Conclusions:**

Our study highlights the unfavorable long-term outcomes in patients with IFDVT who are managed with standard anticoagulation alone. Therefore, consideration of additional strategies may be beneficial to reduce the incidence of post-thrombotic syndrome, and the long-term follow-up is essential to monitor symptom progression.

**Article Highlights**

• **Type of Research:** Single-center retrospective cohort study
• **Key Findings:** At ≥5-year follow-up, reflux and obstruction were present in 57.9% of limbs with iliofemoral deep vein thrombosis (DVT), with 34.2% showing persistent obstruction. Limbs with both reflux and obstruction were significantly more symptomatic and more likely to develop signs of post-thrombotic syndrome than those with a single pathology. Multisegmented DVT was exclusively linked to venous claudication (18.4%), and outcomes were significantly worse than the control limbs.
• **Take Home Message:** Long-term outcomes for iliofemoral DVT treated with anticoagulation alone are poor because of high rates of persistent obstruction and reflux, suggesting that standard medical therapy is insufficient to prevent severe post-thrombotic syndrome.

Thrombus of an iliofemoral (IF) vein accounts for ∼20% to 25% of all lower extremity deep vein thrombosis (DVT).^1^ Iliofemoral DVT (IFDVT) is defined as thrombosis that involves the iliac and/or common femoral veins (CFVs), with or without extension to the inferior vena cava (IVC) or other veins.^2^ IFDVT accounts for 20% to 25% of all lower extremity DVT and is associated with an increased risk of pulmonary embolism, limb malperfusion, and more severe post-thrombotic syndrome (PTS), when compared with less extensive DVT.^3^^,^^4^

IFDVT can lead to prolonged discomfort for patients both physically and socially. Patients may have a range of symptoms, including leg swelling, pain, skin discoloration below the knee, or difficulty walking, with varying degrees of severity.^5^ When DVT recurs it is more likely to cause PTS or worsen clinical symptoms which can greatly impact the quality of life of the patients and result in significant socioeconomic consequences.

Current literature shows a general lack of specificity and uniformity when objectively evaluating the long-term effects of IFDVT. Previous studies have demonstrated that IFDVT is associated with more severe PTS compared with IFDVT, highlighting the importance of especially evaluating long-term outcomes in this subgroup.^6^ In a previous review looking at long-term outcomes, the number of patients and physicians reporting data were low, many patients were lost to follow-up, and signs and symptoms were not consistently defined or reported.^7^ Most studies on IFDVT with or without treatment lack long-term data and have various follow-up. Those with longer term follow-up have significant shortcomings in methodology and execution of the studies.

Our study included patient reported outcomes, physician administered test measures, and objective testing for patients with a first episode of IFDVT treated with anticoagulation having a minimum of 5-year follow-up. The current study was designed to evaluate the long-term natural history of IFDVT when other important contributing factors have been excluded. It also assessed the development of reflux and obstruction and their association with the signs and symptoms using the contralateral limb as a control.

## Methods

This study presents a retrospective analysis of longitudinally gathered data from patients who experienced a first initial episode of IFDVT. Patient data were obtained from an institutional vascular database, which was routinely updated and managed by a trained research principal investigator and vascular surgery team. Institutional Review Board approval was obtained, and informed consent was waived given the retrospective nature of the study. Inclusion criteria were stringent, requiring a minimum of 5 years of follow-up and exclusive use of anticoagulant therapy as the treatment approach. These patients have been under the care of our institution since their initial IFDVT, and they were monitored to observe the natural history of their condition. The study included patients that had thrombosis at least in the CFV or more proximal.

The initial DVT was diagnosed with duplex ultrasound (DU) assessment and computed tomographic venography (CTV). In a few patients, the CTV was performed first, and these patients had also a DU to determine the distribution and extent of thrombosis in all lower limb veins including the iliac veins and IVC. All patients had bilateral testing even in the presence of unilateral symptoms. Patients with IFDVT who were solely treated with anticoagulation were included. They were either treated with low-molecular-weight heparin followed by warfarin or direct oral anticoagulants (apixaban or rivaroxaban), for at least 6 months. Anticoagulation was initiated on the day of diagnosis or within 24 hours in all patients. All patients were recommended to use elastic compression stockings as part of standard management, although compliance was not systematically monitored. Patients were also encouraged to maintain mobility, and limb elevation was encouraged during periods of rest. Patients with any history of pre-existing chronic venous diseases (CVDs) [Clinical, Etiological, Anatomical, and Pathophysiological (CEAP class 2 or higher], previous episodes of DVT, or individuals with peripheral arterial disease, indicated by an ankle-brachial index (ABI) of less than 0.8 and those with a follow-up shorter than 5 years were not included in the study. In addition, patients who had developed an IFDVT due to trauma and subsequently experienced immobility were not part of the study group. We also excluded patients with major comorbidities or systemic and organ failure that could potentially lead to limb swelling or lymphedema. Finally, individuals with restricted mobility, body mass index > 35 kg/m^2^, known cancer or subsequently diagnosed and those with short-life expectancy were not considered for this study.

Concurrently, a control group was created using the contralateral limb. Control limbs included contralateral limbs without DVT, as well as limbs with non-IFDVT (eg, calf, popliteal, or femoropopliteal involvement). In patients with bilateral DVT, each limb was analyzed independently. Limbs were categorized based on the presence or absence of IF involvement. Limbs without IF involvement were included in the control group, even if DVT was present in non-IF segments (eg, calf, popliteal, or femoropopliteal veins). This approach allowed for within-patient comparison while accounting for differences in disease extent.

For each enrolled patient, demographic data, clinical signs and symptoms of CVD, CEAP classification, venous clinical severity score, and episodes of recurrent venous thromboembolism were recorded in detail. Furthermore, on top of the previous imaging testing, a detailed ultrasound examination was also performed at the patient's most recent visit at 5 years or later to assess reflux (from groin to ankle), obstruction and recanalization from IVC to ankle level. The IVC and iliac veins were examined with the patient in supine position with slight torso elevation. To optimize imaging and assessment all infrainguinal veins were examined in the standing position to ensure a physiologic position where the diameter of the veins is larger, that valves are challenged at higher pressure where the presence and duration of reflux are more realistic. Furthermore, the luminal changes can be appreciated better and may show more recanalization as the diameter is larger. When the patient was not able to stand for long time the common femoral, femoral, deep femoral, saphenofemoral junction and the proximal great saphenous vein were evaluated during standing and the rest of the veins in the sitting position. The Jalaie^8^ classification for chronic venous obstruction was used to demonstrate the extent of the disease as this could be the base to predict intervention outcomes.

DU assessment for reflux included standard provocative maneuvers such as distal augmentation and Valsalva where appropriate. Reflux was defined as retrograde flow >1 second in common femoral, femoral, deep femoral and popliteal veins and >0.5 second in the rest of the veins.^9^ Anatomic obstruction was set to be present when the vein was occluded or partially recanalized. Veins that had wall thickening or minimal luminal involvement were considered to be fully recanalized. Patients' demographics were analyzed with descriptive statistics. The symptom development based on various characteristics of IFDVT was calculated using a two-tailed Fisher exact test. As major risk factors were excluded, no analysis was performed for other variables. PTS severity between the IFDVT and control group was compared with the *χ*^2^ test. The likelihood of PTS occurrence was calculated with the odds ratio and 95% confidence intervals (CIs).

## Results

From an original 118 patients, a total of 34 patients (68 limbs) were included in the study. Of these, 38 limbs had IFDVT and comprised the study group, whereas the remaining 30 limbs did not have IF involvement and served as the control group. Analyses were performed at the limb level, and in patients with bilateral DVT, each limb was classified independently based on the presence or absence of IF involvement. The patients included in this study are comprised of 15 males and 19 females. The patients' mean age was 49 ± 11 years, with an age range spanning from 19 to 77 years.

Excluded were 84 patients owing to death before 5-year follow-up (n = 5), short-life expectancy at the time of diagnosis (n = 12), previous DVT (n = 7), CVD (n = 8), ankle-brachial index <0.8 (n = 2), nonvenous edema (n = 2), organ failure (n = 1), refused or unable (n = 13), body mass index >35 kg/m^2^ (n = 2), mobility issues (n = 3), live far or moved (n = 5), neuropathy (n = 1), and vascular malformation (n = 1). The remaining 22 patients were excluded because they did not have a minimum of 5-year follow-up. Of the 34 patients (68 limbs) included, 38 of the limbs had IF involvement, whereas 30 of the limbs did not and comprised the control group. Of the control limbs two had concurrent thrombosis below the CFV and two developed recurrent thrombosis at the popliteal and calf veins. Therefore, 26 limbs of the control group never developed thrombosis.

The 34 patients presented with a range of initial DVT locations. Many of them had also thrombosis in the superficial veins, as outlined in Table I. On the day of diagnosis, 17/34 of the patients (50%) presented with swelling, 2/34 (5.88%) with pain, and 15/34 (44.1%) of them presented with both pain and swelling. Pulmonary embolism signs and symptoms were found in eight patients (23.5%).

**Table I:** Location of deep vein thrombosis (*DVT*)

DVT extent	No. of patients	Concurrent superficial vein thrombosis	No. of patients
IVC + IL	3	GSV	5
IVC + ILFM	10	SSV	2
IVC-POPV	2	GSV + SSV	2
IVC-Calf	2	Nonsaphenous vein	2
IL-POPV	5		
IL-calf	12		
Total	34	Total	11

*FM*, Femoral vein; *GSV*, great saphenous vein; *IL*, iliac vein; *IVC*, inferior vena cava; *POPV*, popliteal vein; *SSV*, small saphenous vein.

Table II shows the occurrence of recurrent ipsilateral DVT for each patient based on the initial extent of their DVT at the 5-year follow-up. Each of these patients could have had either one, two, or three recurrent ipsilateral events. In total there were eight recurrent ipsilateral DVT episodes, and four patients had a contralateral DVT, two of whom was concurrent with the ipsilateral one and in two were on their own. The same patient could have had both an ipsilateral and contralateral DVT.

**Table II:** The presence of deep vein thrombosis (DVT) recurrence based on the initial thrombosis extent

Initial DVT extent	Ipsilateral recurrence			Total	Control limbs		Total (%)
	1×	2×	3×		Concurrent	Separate	
IVC + IL	1	0	0	1		1	
IL/ILFM	1	0	0	1		1	
IVC-POPV	0	0	0	0			
IVC-Calf	1	0	0	1	1		
IL-POPV	0	1	0	2			
IL-calf	0	0	1	3	1		
Total	3	2	3	8	2	2	12 (35.3)

*FM*, Femoral vein; *IL*, iliac vein; *ILFM*, iliac and femoral veins; *IVC*, inferior vena cava; *POPV*, popliteal vein.

Categorical data are shown as number (%).

In addition, the mean Venous Clinical Severity Score at baseline was 7 and the median was 8, with a range spanning from 0 to 18.

Table III provides information on the development of symptoms at 5 years and the classification of patients into CEAP classes based on whether they have isolated or multisegmented DVTs. The odds of presenting with symptoms were significantly lower in patients with isolated DVT compared with those with multisegmented DVT, with an odds ratio of 0.049 (95% CI, 0.005-0.474; *P* = .0035).

**Table III:** Number and characterization of deep vein thrombosis (DVT) of limbs in each Clinical, Etiological, Anatomical, and Pathophysiological (CEAP) class

CEAP class	DVT extent		Symptoms present	
	Isolated	Multisegmented	Isolated	Multisegmented
0-1	4	1	0	1
2	0	2	0	1
3	3	18	1	13
4	0	7	0	6
5	0	2	0	2
6	0	1	0	1
Total	7	31	1	24

At the end of 5 years, edema of varying extent was present in 32 of the 38 limbs (84.2%). The distribution of edema was as follows: eight to the ankle (25%), 11 to the lower calf (34.3%), four to the mid-calf (12.5%), three to the upper calf (9.4%), and six to the thigh (18.7%). Skin damage, those in CEAP class 4 to 6, was found in 26% (10/38) of the limbs. Of the patients with skin damage, three had venous leg ulcers. In addition, 18.4% (7/38) of the limbs developed venous claudication and these were only from those with multisegmented DVTs, all with IF obstruction.

Table IV shows the ultrasound assessment for reflux and obstruction for limbs. The number of segments that were recanalized were also noted. Patients with both reflux and obstruction had significantly higher symptom severity compared to those with either reflux or obstruction alone, with an odds ratio of 11.4 (95% CI, 1.90-68.42; *P* = .0032).

**Table IV:** Ultrasound findings

	Normal	Reflux	Obstruction	Reflux + obstruction	Total
No. of Limbs	2	9	5	22	38
Symptoms present	0	2	3	19	24

	Complete recanalization	Partial recanalization	Occlusion		Total
No. of segments	35	56	31		122

There were eight limbs (21%) with complete recanalization from the CFV up to common iliac vein (CIV). One of these limbs had focal stenosis in the left common iliac vein (LCIV) due to compression from the right common iliac artery. Partial recanalization from CFV to CIV was found in 17 limbs (44.7%). Out of the limbs with only a partial reopening, 11 limbs had long segments of obstruction without an occlusion. The remaining 13 limbs (34.2%) had at least one segment that remained occluded.

Patients either had superficial reflux, perforator reflux, deep vein reflux, or a combination of these. In total, 36.8% (14/38) of the limbs had a deep vein reflux. Superficial and deep vein reflux coexisted in 23.7% (9/38) of the limbs, whereas 18.4% (7/38) of the limbs had a superficial, perforator, and deep vein reflux. No reflux was observed in 13.2% (5/38) of the limbs. Superficial and perforator reflux was present in 2.63% (1/38) of the limbs, whereas 5.26% (2/38) of the limbs had superficial reflux only.

It was shown on the ultrasound study and CTV that five of the patients had a LCIV compression at the acute stage. Focal stenosis of the LCIV was found in another two patients at the chronic stage. Only one patient had a right common iliac vein (RCIV) compression on CTV and ultrasound study that was diagnosed at the acute stage. There was no focal stenosis of the RCIV present even at the chronic stage. However, when looking at the symptom presentation between the left and right leg, there was no statistically significant difference between the two sides (*P* = .729). Based on Jalaie classification^8^ for obstruction seven limbs were normal, one limb had a type 1, 10 had a type 2, 11 had a type 3, 7 had a type 4 (6 with a type 4a and 1 with 4b), and two had type 5.

Table V shows the difference between patients with an IFDVT compared with the uninvolved contralateral limb. Control limbs demonstrated predominantly favorable outcomes at follow-up. Of 30 control limbs, 18 (60%) remained normal, 8 (26.7%) demonstrated reflux, and 4 (13.3%) demonstrated combined reflux and obstruction. No control limbs demonstrated isolated obstruction. Symptoms were present in 9 of 30 control limbs (30%). Limbs with IFDVT are more likely to develop signs and symptoms of PTS, reflux and obstruction, skin damage, and edema compared with those without an initial DVT or those with a DVT that did not have IF involvement. Differences in recurrent DVT between study and control limbs were not statistically significant (χ^2^, 2.377; *P* = .123).

**Table V:** Characteristics (PTS) between patients with affected limbs and unaffected limbs

	Affected limbs (N = 38)	Control limbs (N = 30)	*P* value	OR	95% CI
Signs present	32	11	.0000541	9.21	2.93-28.96
Symptoms present	24	9	.0065985	4	1.44-11.11
Reflux and obstruction present	22	4	.0001737	8.94	2.60-30.70
Skin damage present	10	2	.0348237	5	1.00-24.91
Edema present	32	9	.0000057	12.4	3.86-40.12
DVT recurrence	11	4	.1231132	2.65	0.75-9.38

*CI*, Confidence interval; *DVT*, deep vein thrombosis; *OR*, odds ratio; *PTS*, post-thrombotic syndrome.

VCSS and CEAP scores were also compared between groups and were significantly higher in affected limbs. Affected limbs had higher VCSS scores (mean, 7 vs 1.63; *P* < .001) and a greater proportion of advanced disease (CEAP ≥ 3: 76.3% vs 36.7%; *P* < .001) compared with control limbs.

## Discussion

This article offers distinctive long-term follow-up on the clinical impact of IFDVT for at least 5 years. The existing information has heterogenous data with various degrees of follow-ups, unclear criteria, and lack of objective imaging. This set of data has clinical details and imaging for each patient to help evaluate the long-term outcomes. In addition, each patient was only treated with anticoagulation. Conventional anticoagulant treatment for acute DVT effectively prevents thrombus extension and recurrence, but does not dissolve the clot, and many patients develop PTS.^10^ Without any procedures, it is interesting to note which factors contribute to the severity of PTS in patients. To try to understand the effects of the incident DVT alone, patients with previous DVTs, CVD, and other confounding factors were not included in the analysis, allowing for symptoms to be directly attributed to the DVT itself. In contrast, larger studies such as ATTRACT or CAVENT had more lenient inclusion criteria, allowing patients with previous DVTs or CVD. This makes it more challenging to attribute PTS symptoms solely to the DVT being treated. Notably, CAVENT even included patients with cancer which might have influenced outcomes negatively.^10^^,^^11^ Different from other studies, we used the contralateral leg of the same patient with an IFDVT as a control, allowing for direct comparison and eliminating confounding variables that could occur when comparing different patients.

The main risk factors for PTS are persistent leg symptoms 1 month after an acute DVT, extent of the initial DVT, recurrent ipsilateral DVT, obesity, and older age.^12^ The anatomical extent of venous obstruction and the development of collateral circulation play a significant role in determining the hemodynamic severity of chronic venous obstruction. For example, 11 out of 34 patients had concurrent superficial thrombosis, likely because deep collateral veins are more critical than the superficial venous system in bypassing the obstruction.^13^ Proximal DVT involving the common femoral and/or iliac draining veins, represents a disease process with a worse prognosis and higher risk for poor clinical outcomes compared with proximal DVT sparing these draining veins.^14^ Calf vein involvement in the presence of a proximal DVT has been shown to increase the severity of PTS symptoms.^6^ In our study, patients with an initial DVT extent of iliac vein calf had the highest likelihood of developing ipsilateral recurrence. Recurrent ipsilateral DVT is the next best-established risk factor of PTS after the presence of an IFDVT itself.^11^ Ipsilateral recurrent DVT increases the risk as much as sixfold when compared with patients without recurrence, especially during the initial 3 months of the anticoagulant therapy.^7^^,^^15^

Besides the initial DVT location and ipsilateral recurrence, the extent of segment involvement correlates with the severity of PTS.^6^ Our results also showed that initial multisegmented thrombi lead to a higher percentage of PTS development than single segment thrombi.^6^ Most of the patients in our study fell within CEAP class 3. Within class 3, 72% (13/18) of the patients with multisegmented DVT had symptoms, whereas only 33% (1/3) of the patients with an isolated DVT presented with symptoms. Those in CEAP class 4 to 6, only had patients with an initial multisegmented DVT. Labropoulos et al^6^ demonstrated that venous claudication occurs only in patients with multisegmented DVT and specifically when iliac veins are involved. Our study corroborated these findings, with 18.4% of limbs presenting with venous claudication, exclusively in cases of multisegmented DVT. A recent scoping review further emphasized that venous claudication occurs almost exclusively in patients with IF obstruction, often involving increased venous volume and pressure, compartment edema, and tissue ischemia.^16^^,^^17^

Although obstruction in the absence of reflux is rare, the coexistence of reflux and obstruction is a common finding. Our study found the combination of reflux and obstruction was more predictive of subsequent PTS than the presence of either condition alone, in accordance with the findings of multiple articles.^6^ In our data, 58% of the limbs developed both reflux and obstruction (more than none, reflux alone, and obstruction alone). Among the limbs with reflux and obstruction, 86% developed symptoms. This result corroborates the relationship suggested by Singh and Masuda.^6^ A combination of reflux due to valvular incompetence and venous hypertension due to thrombotic obstruction is thought to contribute to PTS.^18^ Several studies have shown that the rate of recanalization and recurrent thrombotic events are key factors in determining valvular insufficiency. Valvular incompetence plays a significant role in the delayed onset of trophic skin changes and ulceration, commonly seen in PTS.^19^ To summarize, key factors for developing skin damage include the presence of both reflux and obstruction, whereas claudication is more associated with IF obstruction, particularly when the CIV or external iliac veins are occluded. It is also important to note that skin changes, edema, and other symptoms appear to be more directly related to IFDVT than to normal aging, as they are more prevalent when compared with a healthy limb (Table V).

Our research provides further insight into anticoagulation-treated patients, identifying specific PTS risk factors such as ipsilateral recurrence, multisegmented DVT, limbs having both reflux and obstruction over a long period and highlights the importance of ongoing follow-up in these patients. We demonstrated not only the poor long-term outcomes, but also that patients with IFDVT had a higher likelihood of developing PTS compared to their unaffected limb. Based on our findings, patients treated exclusively with anticoagulation had poor long-term outcomes, likely due to moderate-to-severe disease at baseline.

Limitations of our study include the small sample size, absence of physiologic testing, lack of use of validated measures of PTS such as the Villalta scale, the use of a quality-of-life questionnaire and the selection of patients. If iliac stenosis occurs before DVT, the occlusion of the vein may lead to retraction, complicating the distinction of pre-existing retraction from that developing postocclusion. In addition, recanalization success and possible vein narrowing after treatment could influence the impact of iliac stenosis on PTS development.^20^ In our study, focal stenosis was found in only two patients on the LCIV and none in the RCIV. However, there was no significant difference in symptoms presentation among the patients with a left and right-sided DVT. It is possible that the downstream effects of iliac stenosis are underestimated.

The presence of LCIV compression in some patients may suggest contribution to thrombosis development. However, as this study included only patients managed with anticoagulation alone and excluded those undergoing endovascular or surgical intervention, we are unable to determine whether addressing such compression in the acute phase would have altered long-term outcomes.

Although we collected clinical information and imaging, we did not conduct physiologic testing to assess muscle pump function or the overall effect of the thrombosis on the limb. Validated measures such as the Villalta scale and the VEINES-QOL/Sym questionnaire were not routinely used at the time of data collection. Their absence precluded direct comparison with studies such as CAVENT or ATTRACT and to draw conclusions about the personal and social effects of PTS. Additional treatment-related factors that may influence outcomes were not systematically captured. Although all patients were treated with compression therapy, early anticoagulation, and encouraged mobility as part of standard clinical care, compliance and adherence to these measures were not formally monitored. These variables are known to influence venous recanalization and the severity of PTS but could not be incorporated into formal statistical analyses.^21^ Variability in anticoagulation strategies may have also influenced recurrence and recanalization rates; however, the sample size did not permit subgroup analysis of these effects. Future prospective studies with the validated questionnaires and standardized assessment of these factors are warranted.

Furthermore, our stringent exclusion criteria may have compromised our external validity, as the majority of patients with DVT in the real world have comorbidities such as CVD. Selection bias may also be present, as only patients managed without intervention and with available long-term follow-up were included. This may preferentially select for patients more likely to adhere to follow-up and may limit generalizability to the broader DVT population. The smaller sample size from the stringent exclusion criteria also limited the feasibility of multivariable analysis to adjust for potential confounders. Still, inflation of the odds ratio is possible but unlikely to give a false message as the *P* values were very low. These points can be used for future studies.

Nevertheless, our study provides valuable insights into the evolution of IFDVT treated with anticoagulation. Most patients treated with anticoagulation alone experience moderate disease, and a third may develop severe disease (Table III). Therefore, unselected patients may have similar or worse outcomes when taking their comorbidities into consideration.

## Conclusions

Our study highlights the unfavorable long-term outcomes experienced by patients with IFDVT who are treated with anticoagulation alone. The patients in our study had a 5-year follow-up and were carefully selected to include only those with a first episode of DVT with no history of CVD or other factors. We observed that ipsilateral recurrence worsens DVT outcomes and that the presence of multisegmented DVT, along with both reflux and obstruction, results in adverse outcomes such as skin damage and venous claudication. Therefore, new strategies are needed to reduce the incidence of patients with PTS in IFDVT, and long-term follow-up is essential to monitor symptom progression.

## Author Contributions

Conception and design: NL, AG

Analysis and interpretation: NL, AJ, SV

Data collection: NL

Writing the article: NL, AJ

Critical revision of the article: NL, AJ, AG, SV

Final approval of the article: NL, AJ, AG, SV

Statistical analysis: AJ

Obtained funding: Not applicable

Overall responsibility: NL

## Funding

None.

## Disclosures

None.
